# Emergence of the Asian lineage dengue virus type 3 genotype III in Malaysia

**DOI:** 10.1186/s12862-018-1175-4

**Published:** 2018-04-24

**Authors:** Kim-Kee Tan, Nurul-Izzani Zulkifle, Syuhaida Sulaiman, Sui-Ping Pang, NurAsyura NorAmdan, NorAziyah MatRahim, Juraina Abd-Jamil, Meng-Hooi Shu, Nor Muhammad Mahadi, Sazaly AbuBakar

**Affiliations:** 1 Tropical Infectious Diseases Research and Education Centre (TIDREC), University of Malaya, 50603 Kuala Lumpur, Malaysia; 2 Department of Medical Microbiology, Faculty of Medicine, University of Malaya, 50603 Kuala Lumpur, Malaysia; 3 Malaysia Genome Institute, Ministry of Science, Technology and Innovation, Jalan Bangi, 43000 Kajang, Selangor Malaysia

**Keywords:** Infectious disease, Arbovirus, Dengue virus, Malaysia, DENV3, phylogenetic

## Abstract

**Background:**

Dengue virus type 3 genotype III (DENV3/III) is associated with increased number of severe infections when it emerged in the Americas and Asia. We had previously demonstrated that the DENV3/III was introduced into Malaysia in the late 2000s. We investigated the genetic diversity of DENV3/III strains recovered from Malaysia and examined their phylogenetic relationships against other DENV3/III strains isolated globally.

**Results:**

Phylogenetic analysis revealed at least four distinct DENV3/III lineages. Two of the lineages (DENV3/III-B and DENV3/III-C) are current actively circulating whereas the DENV3/III-A and DENV3/III-D were no longer recovered since the 1980s. Selection pressure analysis revealed strong evidence of positive selection on a number of amino acid sites in PrM, E, NS1, NS2a, NS2b, NS3, NS4a, and NS5. The Malaysian DENV3/III isolates recovered in the 1980s (MY.59538/1987) clustered into DENV3/III-B, which was the lineage with cosmopolitan distribution consisting of strains actively circulating in the Americas, Africa, and Asia. The Malaysian isolates recovered after the 2000s clustered within DENV3/III-C. This DENV3/III-C lineage displayed a more restricted geographical distribution and consisted of isolates recovered from Asia, denoted as the Asian lineage. Amino acid variation sites in NS5 (NS5–553I/M, NS5–629 T, and NS5–820E) differentiated the DENV3/III-C from other DENV3 viruses. The codon 629 of NS5 was identified as a positively selected site. While the NS5-698R was identified as unique to the genome of DENV3/III-C3. Phylogeographic results suggested that the recent Malaysian DENV3/III-C was likely to have been introduced from Singapore in 2008 and became endemic. From Malaysia, the virus subsequently spread into Taiwan and Thailand in the early part of the 2010s and later reintroduced into Singapore in 2013.

**Conclusions:**

Distinct clustering of the Malaysian old and new DENV3/III isolates suggests that the currently circulating DENV3/III in Malaysia did not descend directly from the strains recovered during the 1980s. Phylogenetic analyses and common genetic traits in the genome of the strains and those from the neighboring countries suggest that the Malaysian DENV3/III is likely to have been introduced from the neighboring regions. Malaysia, however, serves as one of the sources of the recent regional spread of DENV3/III-C3 within the Asia region.

**Electronic supplementary material:**

The online version of this article (10.1186/s12862-018-1175-4) contains supplementary material, which is available to authorized users.

## Background

Dengue virus (DENV) infection affected approximately 400 million people annually [1]. It is estimated that Asia bore at least 70% of the disease burden [1]. Serologically, DENV can be sub-divided into four distinct serotypes, namely dengue virus type 1 (DENV1), dengue virus type 2 (DENV2), dengue virus type 3 (DENV3), and dengue virus type 4 (DENV4). All four DENV serotypes are able to cause infection in humans through the bite of infected anthropophilic mosquitoes, *Aedes aegypti* and *Aedes albopictus*. Infection with any of the four DENV serotypes presents with similar and indistinguishable clinical manifestations. In general, the infection present with a wide spectrum of clinical manifestations that ranges from asymptomatic, undifferentiated fever to classical dengue fever and a life-threatening severe infection. Even though the four dengue serotypes were shown to be antigenically highly similar [2], infection with one DENV serotype does not confer life-long protection against another DENV serotypes. Transient heterologous immunity, however, can render protection against heterotypic infection for at least three months [3]. Recurring infections are therefore possible and are relatively common especially in hyperendemic regions with co-circulation of multiple DENV serotypes [4, 5].

The widespread distribution of DENV and regional assimilation of virus serotypes and genotypes from close geographical proximity is a reflection of increased regional population mobility and trans-border economic activities [6, 7]. Increasing international travel [8], rapid unplanned urbanization, deforestation, and changing of weather pattern and climate in tropical and sub-tropical regions are among the various factors that could have contributed to the upsurge of dengue during the past 20 to 30 years [8–10]. Continuous importation of DENV into non-traditional dengue regions will likely contribute to the establishment of endemicity in this new region impacting local immune-naïve community [11]. The introduction and subsequent autochthonous transmissions of DENV have been reported in Texas, USA and several European countries, including France, Croatia and Portugal and these served as examples of the continuous spread of dengue outside of the usual dengue endemic regions [12–15]. Epidemiological studies nonetheless, showed that not all but only certain DENV genotypes had wide dispersal to different geographical locations [16, 17]. The DENV3 genotype III (DENV3/III) is one of the DENV genotypes that has been associated with a widespread global distribution [16]. The DENV3/III found in Asia, the Americas, Africa, and Europe appears to originate from the Indian subcontinent and has now become cosmopolitan [16, 18–22]. The virus with these cosmopolitan characteristics demonstrates high transmissibility and is rapidly disseminated upon their introduction to new geographical locations [23].

Here, we report the analysis of the full-length genome of DENV3/III isolates recovered from Malaysia, a dengue hyperendemic country between 1987 and 2012. The genome-scale analyses enabled us to examine the genetic traits, phylogeography, and microevolution of the Malaysian DENV3/IIII strains. Findings from the current study provided insights and a better understanding of the epidemiological characteristics of the DENV3/III subtype recovered from different geographical sites and allowed us to elucidate the possible origin of the recently circulating Malaysian DENV3/III.

## Results

### Global distribution of DENV3 genotype III

**Fig. 1:**
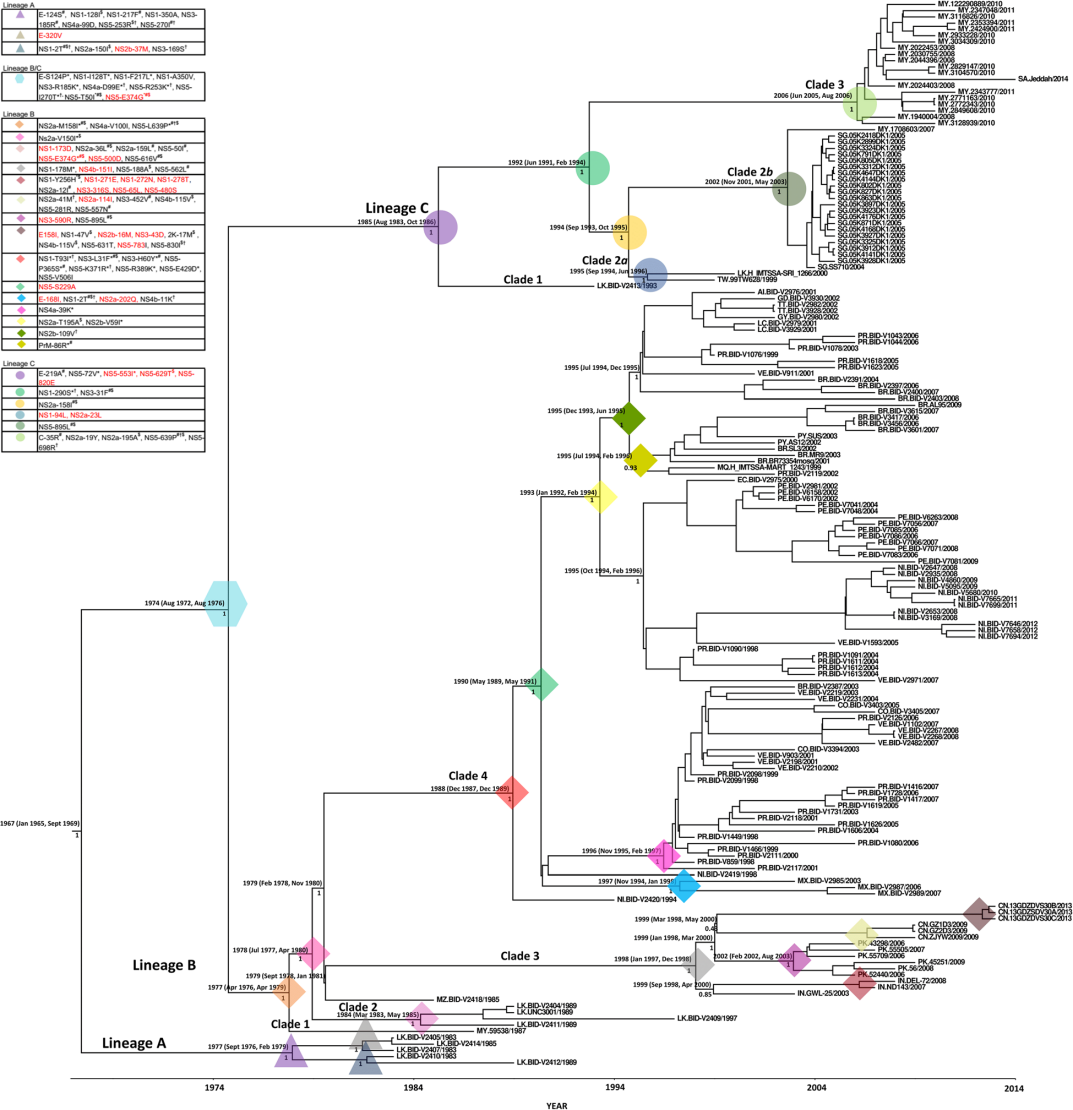
Phylogeny of DENV3/III. The MCC tree of DENV3/III was constructed using complete protein-coding genes. The three lineages of DENV3/III are indicated in the figure: lineages A, B, and C. The estimated 95% HDP values for the MRCA and Bayesian posterior probability values are indicated adjacent to the node. The sequential amino acid substitutions that are inherited by the same group of strains are grouped and assigned with color codes to highlight the isolates that possess the substitutions. The node of which the clade-specific site present is indicated by the color code

For the study, complete coding genome sequences available for DENV3/III isolates reported from 24 countries, and the complete E gene sequences available for isolates from 56 countries between 1966 and 2014 were used (Additional file 1). To gain better spatiotemporal coverage of the DENV3/III strains, we constructed two consensus phylogenetic trees with sequences of the complete coding region (ORF-MCC tree) including the complete genome sequences of 21 newly sequenced Malaysian DENV3/III isolates and those with only complete E gene (E-MCC tree). A total of 602 complete coding sequences (DENV3/III ORF dataset) and 972 E sequences (DENV3/III E dataset) were used for the phylogenetic reconstructions. The RDP4 analysis showed that there was no recombination presence within the ORF and E datasets used in our analysis. With these datasets (ORF and E), the phylogenetic trees suggested that DENV3/III isolates clustered spatially into four major lineages/groups. According to previously suggested nomenclature reported by Messer et al. [16], these lineages were named lineages A, B, C, and D, where lineage A corresponded to group A, while lineage B corresponded to group B. Lineage C (DENV3/III-C) corresponded to group of isolates associated with an unassigned isolate, 93SriLan1 in the same study [16]. Lastly, the lineage D represented a group of isolates (IN.NIV_664481/1966 and IN.INV_664482/1966 and one Samoa isolate, WS.1696/1984) recovered before the 1990s. These isolates, however, segregated into three lineages on the ORF-MCC tree that corresponded to lineages A, B, and C on the E-MCC tree. This was because of the complete genome sequences of the two Indian isolates, IN.NIV_664481/1966 and IN.INV_664482/1966 and one Samoa isolate, WS.1696/1984 that made-up the lineage D on the E-MCC tree were not available. Overall, the clustering of the overlapped isolates on the ORF-MCC tree and E-MCC tree was consistent. For simplicity of presentation, we sub-sampled a total of 169 representative isolates covering the full range of genetic diversity of DENV3/III observed in the initial phylogenetic tree. This subsampled dataset was used to construct a second phylogenetic tree (Fig. 1; A copy of full E-MCC tree and ORF-MCC are available upon request from the corresponding author). The sequential amino acid substitutions derived from the entire protein-coding region were determined. Informative sequential amino acid substitutions and clade-specific variation sites were summarized and incorporated into the MCC tree (Fig. 1).

**Fig. 2:**
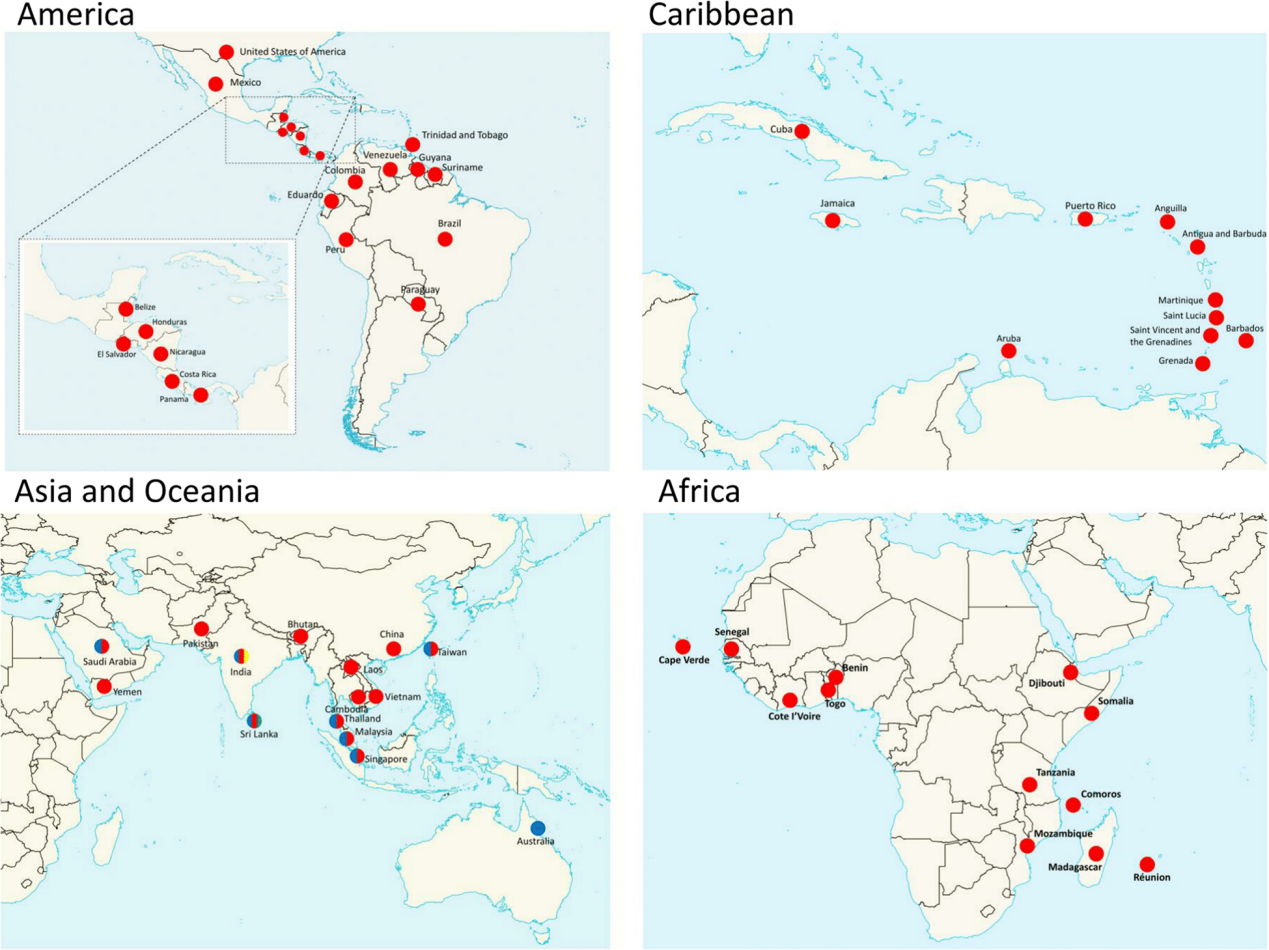
Geographical distribution of DENV3/III. The colored circles indicate the isolation of different DENV3/III lineages where green represents DENV3/III-A, red represents DENV3/III-B, blue represents DENV3/III-C, and yellow represents DENV3/III-D. The map was created and modified from Natural Earth, free vector and raster map data @ naturalearthdata.co using QGIS Desktop 2.16.2. The map is licensed under Creative Commons Attribution-ShareAlike 3.0 Licence (CC BY-SA)

To date, at least fifty-six countries across the Americas, Caribbean, Africa, Asia, and Oceania regions have reported dengue cases caused by DENV3/III (Fig. 2). The viruses were recovered from all parts of the Americas: South America (Brazil, Colombia, Eduardo, Guyana, Peru, Paraguay, Suriname, Trinidad and Tobago, and Venezuela), Central America (Belize, Costa Rico, Honduras, Nicaragua, Panama, and El Salvador), and North America (Mexico and United States of America). Eleven countries in the Caribbean region: Antigua and Barbuda, Anguilla, Aruba, Barbados, Cuba, Grenada, Jamaica, Saint Lucia, Martinique, Puerto Rico, and Saint Vincent and the Grenadines reported the isolation of the DENV3/III. DENV3/III was also detected in all parts of Asia: East Asia (China and Taiwan), South Asia (Bhutan, India, Pakistan, and Sri Lanka), SEA (Cambodia, Laos, Malaysia, Singapore, Thailand, and Vietnam), and West Asia (Saudi Arabia and Yemen) and several countries in the African region: East Africa (Djibouti, Mozambique, Somalia, and Tanzania), South Africa (Comoros and Madagascar), and West Africa (Benin, Cape Verde, Cote d’Ivoire, Senegal, and Togo). Lastly, the DENV3/III was also recovered in Oceania region: Australia, French Polynesia, and Samoa.

### Selection pressure analysis of the complete coding region of DENV3 genotype III

The selection pressure analysis of the complete coding region of DENV3/III was performed using the HyPhy package as implemented in Datamonkey server [24]. A combination of codon-based selection pressure analysis (SLAC, FEL, and IFEL) with Hierarchical Bayesian based analysis (FUBAR) and Branch-site based analysis (MEME) revealed that 19 sites in the entire complete coding region of DENV3/III were under positive selection (Table 1). The positive selection sites were identified in PrM, E, NS1, NS2a, NS2b, NS3, NS4a, and NS5. No sites were inferred as under positive selection for C, NS4b, and 2 K genes. Of the 19 positively selected sites, seven sites were located on the structural genes, while the remaining 12 sites were located on the non-structural genes.

**Table 1:** List of positive selection sites of DENV3/III isolates

Dataset	Codon	SLAC		FEL		IFEL		MEME		FUBAR	
		dN-dS	*p*-value	dN-dS	*p*-value	dN-dS	*p*-value	ω^+^	*p*-value	dN-dS	Post. Pr.
PrM (*n* = 239)	34	−0.1076	0.7553	0.0259	0.9761	−0.7189	0.3047	> 100	0.0072	−0.0217	0.4180
	49	−2.6595	0.8733	−1.1322	0.3360	− 1.7419	0.0866	> 100	0.0172	−0.1367	0.2155
ENV (*n* = 413)	**132**	3.9013	0.0459	3.5956	0.0011	2.3190	0.0265	> 100	0.0123	1.0099	0.9926
	169	1.4647	0.2964	0.7594	0.1001	0.0000	1.0000	> 100	0.0002	0.1242	0.7448
	**329**	4.8826	0.0385	2.1726	0.2442	3.9530	0.1060	5.7227	0.0488	0.9124	0.9587
	**380**	2.4094	0.1406	1.4397	0.0272	1.5090	0.0486	> 100	0.1017	0.2447	0.8887
	404	−1.7093	0.9383	−1.0008	0.2073	−1.2489	0.0955	> 100	0.0003	−0.3071	0.0592
NS1 (*n* = 364)	17	−4.4925	0.9670	−0.2710	0.7483	−1.0741	0.1907	> 100	0.0000	−0.1041	0.2427
	178	2.0170	0.4206	−0.0837	0.9395	0.9296	0.5599	> 100	0.0268	0.0040	0.4414
NS2a (*n* = 314)	**210**	5.7384	0.1322	3.0481	0.0176	1.7719	0.1963	4.0752	0.2027	0.7751	0.9886
NS2b (*n* = 193)	73	−4.7928	0.9710	−1.2591	0.2573	−1.9587	0.0990	> 100	0.0109	−0.1473	0.1410
	104	−0.7126	0.7272	−0.9021	0.3749	−1.7010	0.1328	71.8889	0.0314	−0.0798	0.2094
NS3 (*n* = 435)	278	−6.2702	0.9997	−2.1476	0.0550	−2.6032	0.0536	> 100	0.0140	−0.8322	0.0130
	**338**	3.1369	0.0958	2.5345	0.0291	2.2087	0.0703	> 100	0.0401	0.5927	0.9305
	379	−3.1511	0.9800	−0.9745	0.3703	−1.5012	0.1476	> 100	0.0002	−0.3206	0.1092
NS4a (*n* = 205)	15	3.9699	0.1877	0.6802	0.2249	1.6358	0.0839	> 100	0.1013	0.0468	0.6667
NS5 (*n* = 472)	616	3.8130	0.1317	1.3480	0.0316	1.4558	0.0514	> 100	0.1057	0.2726	0.8640
	629	3.9319	0.1246	1.1144	0.2054	1.2144	0.2130	> 100	0.0487	0.1290	0.6829
	700	2.5290	0.9930	0.9094	0.9531	0.0000	1.0000	> 100	0.0025	−1.2759	0.3429

The positively selected site that identified by more than one methods was bold

Three (codons 132, 329, and 380) out of the five positively selected sites in E gene were identified simultaneously by more than one methods. Among the three sites, the E-132 was identified as positively selected sites by all five methods employed in the current analysis (SLAC: *p*-value = 0.0459, FEL: *p*-value = 0.0011, IFEL: *p*-value = 0.0265, MEME: *p*-value = 0.0123, and FUBAR, pp. = 0.9926). The E-329 was positive by SLAC (*p*-value = 0.0385), MEME (*p*-value = 0.0488), and FUBAR (pp = 0.9587). The E-380 was identified as positive selection site by FEL (*p*-value = 0.0272) and IFEL (p-value = 0.0486). The remaining two positively selected sites in E, the E-169 (*p*-value = 0.0002) and E-404 (*p*-value = 0.0003) were identified by MEME.

As for the non-structural genes, two positively selected sites were identified simultaneously by more than one methods. The NS2a-210 were identified by FEL (*p*-value = 0.0176 and FUBAR (pp = 0.9886). The NS3–338 was identified as positively selected sites by three methods used in the analysis (FEL: *p*-value = 0.0291, MEME: *p*-value = 0.0401, and FUBAR: pp. = 0.9305). The NS3–338 was positive (*p* value between 0.05 and 0.1) by SLAC (*p*-value = 0.0958) and IFEL (*p*-value = 0.0703).

### Molecular signature and phylogenetic relationship of DENV3 genotype III

Phylogenetically, the DENV3/III-A, -B, and -C were occupied by DENV3/III strains isolated during the 1980s (Fig. 1). The MRCA analysis showed that the common ancestor of lineage A/B/C could have circulated in 1967 (95% HDP: 1965 to 1969). Whereas, the split of the MRCA of the lineage B/C viruses dated back to 1974 (95% HDP: 1972 to 1976). No DENV3/III-D (data not shown) and DENV3/III-A strains were recovered after its last recorded isolation in the 1980s. DENV3/III-B and DENV3/III-C, on the other hand, exhibited greater diversification and were sustained in the populations until today. The Malaysian DENV3/III viruses sequenced in our study clustered within DENV3/III-B and DENV3/III-C.

There was a strong spatial and temporal clustering pattern within DENV3/III-B isolates. A single Malaysian isolate (MY.59539.1987) recovered in the year 1987 occupying the ancestral node of DENV3/III-B. Herein, the MY.59538/1987 was denoted as clade 1 (DENV3/III-B1). The DENV3 strains recovered in Sri Lanka during late of the 1980s and early 1990s were denoted as clade 2 (DENV3/III-B2). While the strains recovered in India (2003–2008), China (2009–2013), and Pakistan (2006–2009) clustered into clade 3 (DENV3/III-B3). The DENV3/III-B clade 4 (DENV3/III-B4) consisted of isolates recovered in Latin America. The MRCA analysis demonstrated that the circulation of ancestral strains of DENV3/III-B dated back to the late 1970s (95% HDP: 1976 to 1979). The DENV3/III-B lineage diverged rapidly and gave rise to DENV3/III-B2 and DENV3/III-B3/4 viruses around 1977 to 1980 (95% HDP), followed by the split of DENV3/III-B3 and DENV3/III-B4 viruses that dated back to 1978 to 1980 (95% HDP). The analysis of the deduced amino acid substitutions revealed the presence of one amino acid substitution where valine was substituted with isoleucine in the NS4a (NS4a-100) that was shared by the DENV3/III-B viruses. The DENV3/III-B clade2/3/4 strains shared a specific amino acid substitution where the valine at the position of 150 of NS2a proteins was substituted by isoleucine.

The other Malaysian DENV3/III isolates (2007–2011), however, fell into another lineage, the DENV3/III-C (Fig. 1). The ancestral node of the DENV3/III-C was occupied by a Sri Lankan isolate, LK.BID-V2413/1993 recovered in Sri Lanka in 1993. The DENV3/III-C viruses shared five common amino acid variations: E-219A, NS5-72V, NS5–553I, NS5–629T, and NS5–820E. Among the five amino acid variation sites, the NS5–553I, NS5–629T, and NS5–820E were unique to DENV3/III-C viruses. These five amino acid substitutions were probably present in the ancestral strain pool that was circulating in the region approximately 30 years ago (95% HDP: 1983 to 1986), which was prior to the diversification of DENV3/III-C viruses.

**Fig. 3:**
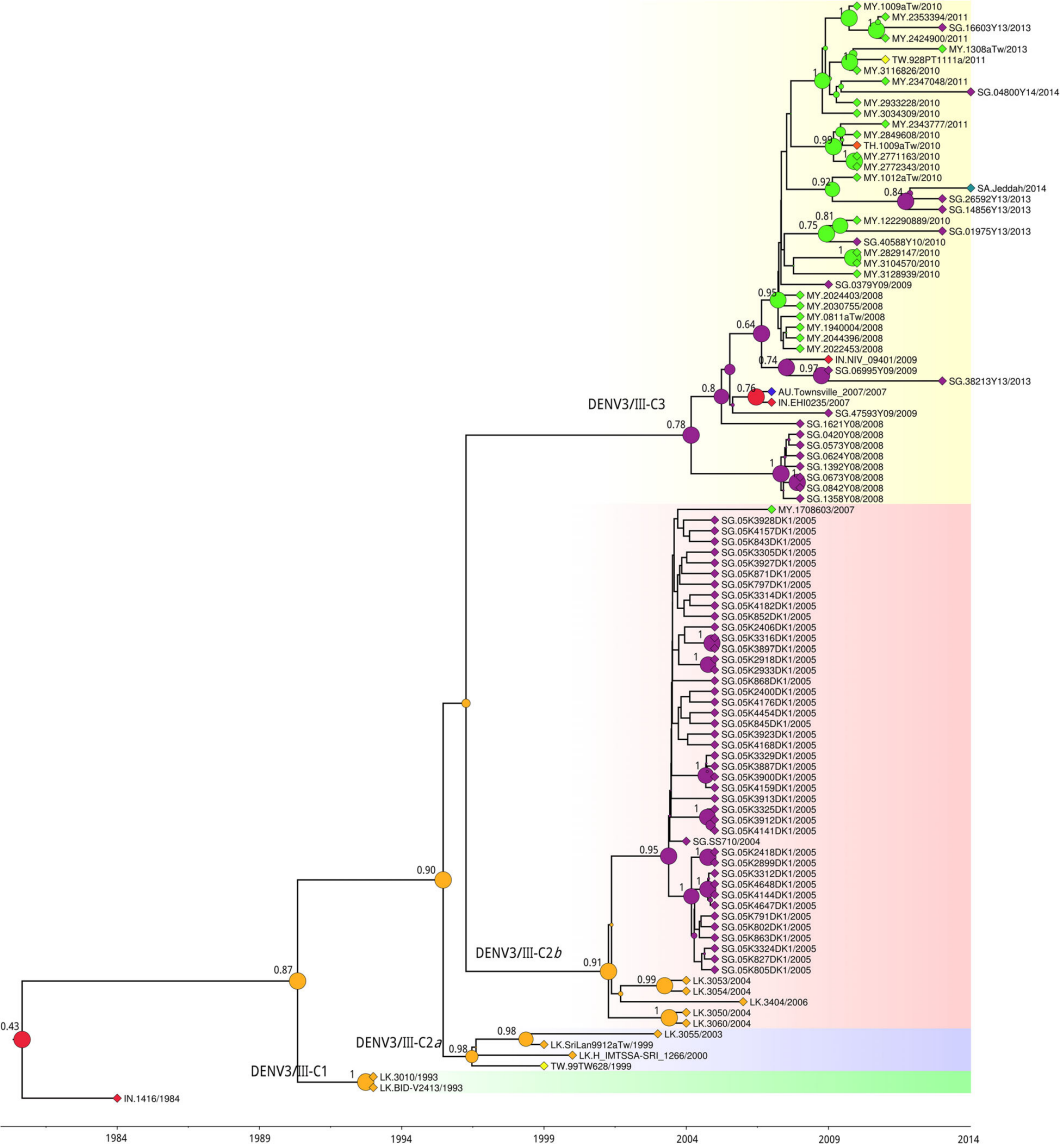
Phylogeography of DENV3/III-C. The MCC tree of DENV3/III-C was constructed using the complete E gene of 102 DENV3/III-C strains. The different DENV3/III-C clades are indicated in this figure with pink for DENV3/III-C1, green for DENV3/III-C2*a*, blue for DENV3/III-C2*b*, and gold for DENV3/III-C3. The locations where the DENV3/III-C strains were recovered are indicated with the colored diamond shape at the branch tip while the estimated ancestral location states of each internal branch are shown with colored circles on the branch node. The blue diamond/circle represents Australia, red diamond/circle represents India, light orange diamond/circle represents Sri Lanka, green diamond/circle represents Malaysia, turquoise diamond/circle represents Saudi Arabia, purple diamond/circle represents Singapore, orange diamond/circle represents Thailand, and yellow diamond/circle represents Taiwan. The size of node circle is proportionate to the posterior value of the node. The estimated location probability values for an internal node with posterior value more than 0.7 are indicated adjacent to the node

Within DENV3/III-C, isolates were segregated temporally into three monophyletic clades (Fig. 1 and Fig. 3). At the basal node of the DENV3/III-C clade 1 (DENV3/III-C1) were viruses isolated from Sri Lanka in 1993 (Figs. 1 and 3). No virus from this lineage was isolated for the next six years until the isolation of DENV3/III-C2 strains in 1999. All of the DENV3/III-C2 viruses shared amino acid substitutions of isoleucine at position 158 of NS2a protein (Fig. 1). Within DENV3/III-C2, the viruses were segregated into two subclades. The DENV3/III-C2 subclade *a* (DENV3/III-C2*a*) consisted of strains recovered in Sri Lanka and Taiwan recovered during 1999 to 2000. The DENV3/III-C2*a* viruses (TW.99TW628/1999 and LK.H_IMTSSA-Sri_1266/2000) shared two unique and specific amino acid variation sites: NS1-94L and NS2a-23L. The NS1-94L/NS2a-23L-bearing ancestral pool was probably circulating in Sri Lanka during 1994 to 1996 (95% HDP). Analysis of the DENV3/III E-MCC tree showed an additional isolate, the LK.SriLan9912aTW/1999 isolated in Taiwan with Sri Lanka origin which clustered in DENV3/III/C2*a* (Fig. 3). The DENV3/III-C2 subclade *b* (DENV3/III-C2*b*) referred to a group of Singaporean isolates recovered during 2004 to 2005 and a single Malaysian DENV3/III, MY.1708603/2007 recovered in 2007. For the DENV3/III-C2*b* viruses, an amino acid variation of leucine at position 895 of NS5 protein was observed in the genome of all its descendants. The NS5-895L-bearing strains were most probably appeared or introduced into the regions in the early of 2000 (95% HDP: 2001 to 2003), which was three years prior to the isolation of the first virus, SG.SS710/2004 in Singapore. There was no isolate from subclade *a* and subclade *b* detected after 2003 and 2007, respectively. All DENV3/III isolates recovered from Malaysia, Singapore, and Thailand after 2007 grouped and formed a new clade, DENV3/III-C3 (Fig. 1 and Fig. 3). Based on the phylogenetic tree, this DENV3/III-C3 was not the direct descendant of the DENV3/III-C2 viruses that were circulating in Singapore in 2004 to 2005. The DENV3/III-C2 and DENV3/III-C3 likely diverged in 1992 (95% HDP: 1991 to 1994). The DENV3/III-C3 viruses carried a combination of five amino acid substitutions: C-35R, NS2a-19Y, NS2a-195A, NS5-639P, and NS5-698R. The NS5-698R was a unique amino acid site to DENV3/III-C3.

### Phylogeography of DENV3 genotype III lineage C

In order to investigate the potential spread of DENV3/III-C, we reconstructed the ancestral geographic state of the DENV3/III-C (Fig. 3 and Additional file 2). The phylogenetic tree of the DENV3/III-C branch demonstrated a ladder-pattern, suggesting the virus was likely to have originated from Sri Lanka (Loc. Prob = 0.87) and spread into Taiwan (DENV3/III-C2*a*, Loc. Prob = 0.98). Similarly, the DENV3/III-C2*b* had an ancestor that was likely to have originated from Sri Lanka (Loc. Prob = 0.91). Our results, however, demonstrated that the DENV3/III-C3 originated from Singapore (Loc. Prob = 0.78). A group of Singaporean isolates recovered in 2008 occupied the basal node of DENV3/III-C3 on the E-MCC tree, followed by isolates recovered in India (2007), Australia (2007), and Singapore (2008 to 2009). The Malaysian DENV3/IIIC3 formed a monophyletic clade within DENV3/III-C3. Isolates collected from Thailand (TH.1009aTw/2010), Taiwan (TW.928PT1111a/2011), Saudi Arabia (SA.Jeddah/2014), and Singapore (SG.40588Y10/2010, SG.01975Y13/2013, SG.0379Y09/2009, SG.14856Y13/2013, SG.26592Y13/2013, SG.16603Y13/2013, and SG.04800Y14/2014) clustered and interspersed within the Malaysia DENV3/III-C3 monophyletic clade. Our results suggested that the Thai (TH.1009aTw/2010), Taiwanese (TW.928PT1111a/2011), and a few Singaporean strains were likely to have been introduced from Malaysia. The Saudi Arabian strain, SA.Jeddah/2014, however, most likely originated from Singapore.

### Molecular signature of Malaysian DENV3 genotype III lineage C3

Pairwise comparison of the Malaysian DENV3/III strains recovered from 2008 to 2011 revealed the presence of 248 nucleotide substitutions, resulting in a genetic diversity of ~ 2.4%. These nucleotide substitutions were translated into 36 amino acid substitutions (Table 2), of which 10 were located on the structural genes (C and E), while the remaining 26 amino acid variations were located on the non-structural genes (NS1, NS2a, NS2b, NS3, NS4a, 2K, NS4b and NS5). No amino acid variation was observed in PrM. These amino acid variations resulted in ~ 1.06% amino acid changes. Thirteen out of the 36 amino acid variations were characterized as parsimony informative sites, where the variations were identified in the genome of at least two DENV3/III strains. Based on the parsimony informative sites, the amino acid variation at NS3 gene at position 506 (isoleucine or leucine) could discriminate the Malaysian DENV3/III-C3 into two subgroups, and each subgroup consisted of isolates recovered from 2008 to 2011. The subsequent 12 parsimony informative sites, eight (E-L234R, E-V249A, E-T471I, NS3-K15R, 2K-L17M, NS4a-K12R, NS5-D808E, and NS5-N835D) were identified within the NS3-506I-bearing subgroup. Whereas, the remaining four variation sites, C-L105F, E-E79D, NS4b-S23P, and NS5-P636S were identified among the NS3-506L-bearing subgroup.

**Table 2:** Amino acid substitutions identified between the Malaysian DENV3/III-C3 isolates recovered between 2008 to 2011

Virus strains	C			E							NS1		NS2a	NS2b	NS3								NS4a		2 K	NS4b		NS5								
	65	76	105	50	51	79	234	249	471	482	286	290	120	91	15	60	120	142	468	419	506	585	2	19	17	17	23	24	155	288	374	591	636	735	808	835
MY.1940004/2008	V	G	L	A	T	E	L	V	T	I	V	S	I	T	K	H	T	R	A	K	I	K	I	H	L	K	S	E	T	N	E	I	P	I	D	N
MY.3128939/2010	.	.	.	.	.	.	.	.	.	L	.	.	.	.	.	.	.	.	T	.	.	.	.	Y	.	.	.	.	.	.	.	.	.	.	.	D
MY.2343777/2011	.	.	.	.	.	.	.	.	I	.	.	.	F	.	R	.	.	.	.	.	.	R	.	.	M	R	.	.	.	.	G	.	.	.	.	D
MY.2772343/2010	.	.	.	.	.	.	R	A	I	.	.	.	.	.	R	.	.	.	.	.	.	.	.	.	M	R	.	.	.	.	.	.	.	.	E	.
MY.2771163/2010	.	.	.	.	.	.	R	A	I	.	.	.	.	.	R	.	.	.	.	.	.	.	.	.	M	R	.	.	.	.	.	.	.	.	E	.
MY.2849608/2010	.	R	.	.	.	.	.	.	I	.	.	.	.	.	R	.	.	.	.	.	.	.	.	.	M	R	.	.	.	.	.	.	.	V	.	.
MY.2024403/2008	.	.	.	.	.	.	.	.	.	.	.	.	.	.	.	.	.	.	.	.	.	.	.	.	.	.	.	.	.	.	.	.	.	.	.	.
MY.2022453/2008	.	.	.	.	.	.	.	.	.	.	.	.	.	.	.	.	.	.	.	.	L	.	.	.	.	.	.	.	.	.	.	.	.	.	.	.
MY.122290889/2010	.	.	.	.	.	.	.	.	.	.	.	.	.	.	.	.	A	.	.	R	L	.	.	.	.	.	.	D	.	.	.	.	.	.	.	.
MY.3034309/2010	.	.	.	V	.	.	.	.	.	.	.	.	.	.	.	Y	.	.	.	.	L	.	.	.	.	.	.	.	.	D	.	.	.	.	.	.
MY.2933228/2010	.	.	.	.	.	.	.	.	.	.	.	.	.	A	.	.	.	.	.	.	L	.	.	.	.	.	.	.	.	.	.	.	.	.	.	.
MY.2424900/2011	.	.	.	.	.	.	.	.	.	.	.	.	.	.	.	.	.	.	.	.	L	.	.	.	.	.	P	.	I	.	.	.	.	.	.	.
MY.2353394/2011	.	.	.	.	.	.	.	.	.	.	.	.	.	.	.	.	.	K	.	.	L	.	.	.	.	.	P	.	.	.	.	.	.	.	.	.
MY.3116826/2010	.	.	.	.	.	.	.	.	.	.	.	.	.	.	.	.	.	.	.	.	L	.	.	.	.	.	.	.	.	.	.	V	.	.	.	.
MY.2347048/2011	I	.	.	.	.	.	.	.	.	.	I	N	.	.	.	.	.	.	.	.	L	.	V	.	.	.	.	.	.	.	.	.	.	.	.	.
MY.2044396/2008	.	.	.	.	.	.	.	.	.	.	.	.	.	.	.	.	.	.	.	.	L	.	.	.	.	.	.	.	.	.	.	.	.	.	.	.
MY.2030755/2008	.	.	.	.	I	.	.	.	.	.	.	.	.	.	.	.	.	.	.	.	L	.	.	.	.	.	.	.	.	.	.	.	.	.	.	.
MY.3104570/2010	.	.	F	.	.	D	.	.	.	.	.	.	.	.	.	.	.	.	.	.	L	.	.	.	.	.	.	.	.	.	.	.	S	.	.	.
MY.2829147/2010	.	.	F	.	.	D	.	.	.	.	.	.	.	.	.	.	.	.	.	.	L	.	.	.	.	.	.	.	.	.	.	.	S	.	.	.

## Discussion

The emergence of DENV3/III and its geographical dispersal have been described in many studies over the past two decades [16, 20, 22, 25, 26]. These newly emerged DENV3/III strains were usually associated with strains originated from Sri Lanka, in particular, the DENV3 that caused the 1989 DHF outbreak (group B) [7, 21, 27]. Detailed phylogenetic and traceable amino acid substitutions analysis revealed that the currently circulating DENV3/III strains emerged from at least two major groups of founding viruses that descended from an ancestral strain of old DENV3/III that diverged between 1972 to 1976 (95% HDP). Our findings were consistent with others [16] suggesting that one of the current actively circulating DENV3/III lineages corresponded to the previously described DENV3/III group B viruses, herein denoted as DENV3/III-B. The other current actively circulating DENV3/III was associated with the uncharacterized DENV3 strain, 93SriLan1 that was initially described in the same study [16], herein denoted as DENV3/III-C. Our findings suggested that both the actively circulating DENV3/III lineages originated from the Indian subcontinent regions. The founding viruses of DENV3/III-B and DENV3/III-C could have diverged and dispersed out from the Indian subcontinent through independent wave-like events [16] after the accumulation of a combination of ten amino acid substitutions shared within the genome of DENV3/III-B and DENV3/III-C viruses. The DENV3/III-B has a cosmopolitan distribution and represent those viruses that spread into regions including East Africa, Sri Lanka, and other American regions and territories during the first wave of dispersal which started in the 1980s [16, 28, 29]. This lineage continues to evolve and spread to present locations [18, 22, 30, 31]. We reported for the first time possible involvement of SEA particularly Malaysia during the spread of DENV3/III in the 1980s [16]. The isolation of MY.59538/1987 in 1987 coincided temporally with the global spread of DENV3/III described by Messer et al. (first wave of DENV3/III spread). Due to the limited number of old Malaysian DENV3/III isolates, however, we could not rule out the possibility that MY.59538/1987 was an imported isolate. More samples are needed to allow a better understanding of the possible involvement of Malaysia during the spread of DENV3/III-B at the end of the 1980s. Nonetheless, no other DENV3/III-B was isolated from any SEA countries during the early phase of virus spread in the 1980s except MY.59538/1987 described in the current study, suggesting possible transient introduction into the SEA during the early stage of the first wave of DENV3/III spread. Whereas, the spread of DENV3/III-C representing the second wave of DENV3/III dispersal from the same origin (Indian subcontinent region) likely started within the past two decades (tMRCA = 1994). When compared to DENV3/III-B, the DENV3/III-C has a more restricted geographical distribution that narrowly focused to Asia, hence, denoted as the DENV3/III Asian lineage. The subsequent geographical and temporal diversification of viral populations within DENV3/III-B and -C reflects that the viruses continue to evolve independently and spread after the point of segregation.

The MY.59538/1987 was the only DENV3 isolated in Malaysia had been assigned as DENV3/III-B. The recent DENV3/III isolates recovered in Malaysia were clustered within DENV3/III-C. The clear segregation of the old (MY.59538/1987) and recent Malaysian DENV3/III isolates, suggesting that the newly emerged Malaysian DENV3/III (DENV3/III-C) isolates were not the direct descendant of the old Malaysian DENV3/III. The MY.59538/1987 which clustered in DENV3/III-B lineage had a closer phylogenetic relationship with isolates currently circulating in the Americas and Asia but not with those that were recently recovered in Malaysia. Phylogenetic analysis revealed a close relationship of the newly emerged Malaysian DENV3/III with other DENV3/III isolates recovered from Singapore [7] and Taiwan (Thailand-origin) [32] (DENV3/III-C), but not with DENV3/III strains recovered from Japan (Cambodia-origin) [33], Laos [34], and China [18, 35] (DENV3/III-B). Considering the proximity and geo-distribution and distinct phylogenetic relationships of DENV3/III recovered from the North-Western (Cambodia, Laos, and China) and South-Eastern (India, Thailand, Malaysia, and Singapore) part of Asia, our results herein suggested that there were at least two distinct yet concurrent regional transmission routes for DENV3/III within Asia. So far, only the DENV3/III-C transmission route involved Klang Valley, Malaysia.

**Fig. 4:**
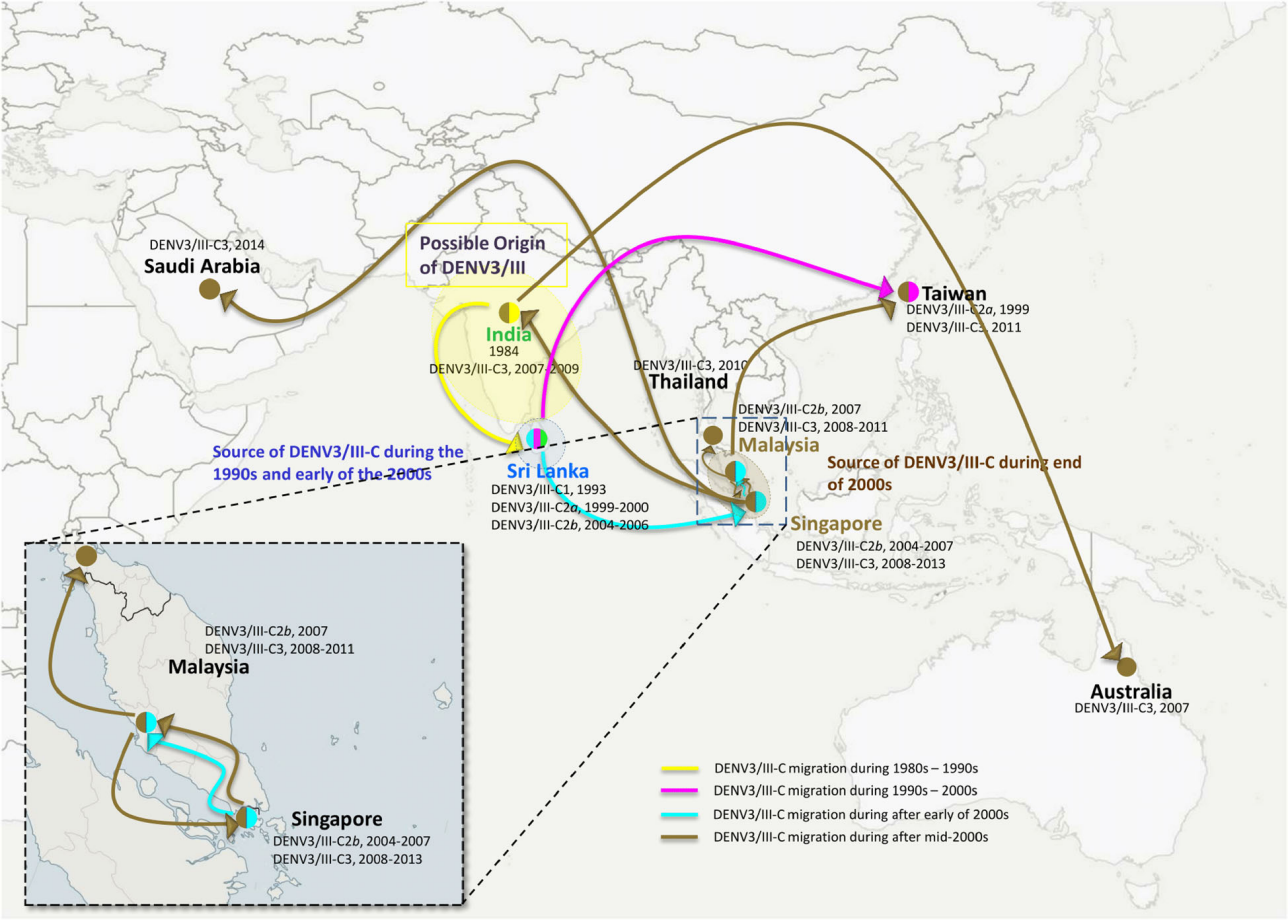
Spread of DENV3/III-C. The movement of Asian lineage of DENV3/III (DENV3/III-C) in Asia and Oceania region. The colored circles indicate the isolation of different DENV3/III-C lineages. Yellow represents isolate collected before 1990, green represents DENV3/III-C1, pink represents DENV3/III-C2*a*, cyan represents DENV3/III-C2*b*, and gold represents DENV3/III-C3. The map was created and modified from Natural Earth, free vector and raster map data @ naturalearthdata.co using QGIS Desktop 2.16.2. The map is licensed under Creative Commons Attribution-ShareAlike 3.0 Licence (CC BY-SA)

Microevolution of the DENV3/III-C genome integrated into our phylogeography analysis allowed us to reconstruct the possible transmission route of DENV3/III-C in Asia. Our findings suggested that the DENV3/III-C evolved from a single common ancestral node, highlighting the possibility that the DENV3/III-C emerged from a single origin in the Indian subcontinent (Fig. 4) after the accumulation of a combination of five amino acid substitutions in the genome of the founding virus strain. Among these five DENV3/III-C amino acid substitutions, three (NS5–553, NS5–629, and NS5–820) were lineage-specific mutations that allowed differentiation of DENV3/III-C from other DENV3/III viruses. These three DENV3/III-C specific mutations were located in RNA-dependent RNA polymerase domain. The codon 629 located in the palm subdomain, most structurally conserved subdomain of the NS5 protein [36], underwent positive selection (*p* < 0.05). Whether these naturally occurred mutations in the NS5 protein of DENV3/III-C would have an impact on the virus fitness of the DENV3/III-C, warrant further investigation. Prior to the emergence of DENV3/III-C in Singapore during 2003, the isolation of DENV3/III-C was random and did not associate with any outbreak or endemic circulation at a single locality [37, 38] except for the endemic circulation in Sri Lanka revealed from the current study. During the 1990s, there were only three DENV3/III-C isolates recovered from Sri Lanka and Taiwan (DENV3/III-C2). Continuous isolation of the Sri Lankan isolates from 1993 to 2006 [39], is suggestive of persistent circulation of DENV3/III-C in the local setting of Sri Lanka and possibly the Indian subcontinent region [40]. The DENV3/III-C was likely to circulate with low transmission level in comparison to other dominant DENV3 lineages, the DENV3/III-A (pre-1989 DHF epidemic predominant strains) and DENV3/III-B (1989 DHF outbreak-causing strain in Sri Lanka) [16]. Whereas, the isolation of the virus in 1999 from a Taiwan indigenous individual who did not have travel history, suggested an earlier unrecorded eastern spread of the DENV3/III-C virus into Taiwan [38] from Sri Lanka (Fig. 4). The emergence of DENV3/III-C in Singapore beginning from 2003 and isolation of DENV3/III-C2*b* in Malaysia in 2007 (MY.1708603/2007) were additional evidence for the eastward dispersal of the virus [21] from Sri Lanka. Our results showed that the MY.1708603/2007, the only Malaysian DENV3/III-C2*b* recovered so far was likely a random imported case from Singapore.

The 2004–2007 Singapore and Malaysia DENV3/III-C strains (DENV3/III-C2*b*) clustered closely and distinctly from the DENV3/III-C strains recovered from the same geographical locations (Malaysia and Singapore) after 2008 (DENV3/III-C3). This was consistent with the report by Lee et al. [7], which showed that the DENV3/III-C recovered from the SEA after 2008 formed a new clade. The DENV3/III-C2*b* consisted of Singapore 2004–2007 viruses, was completely replaced and became extinct by 2008. The finding hence, suggests that DENV3/III-C3 viruses isolated from SEA countries were not likely to have emerged from viruses that were locally circulating during 2004 to 2007. Phylogenetic analysis revealed the presence of one monophyletic Singapore cluster located at the ancestral node of DENV3/III-C3. Other isolates recovered from Malaysia, India, and Australia were interspersed within the clade descendent from the 2008-Singaporean clade. The first isolation of the most recent DENV3/III-C from Malaysia in 2008, Thailand in 2010, Taiwan in 2011, and Saudi Arabia in 2014, suggested that the viruses continue to evolve and spread regionally after their introduction into the regions, probably through founding viruses that circulated in Singapore [32]. The DENV3/III-C3, however, did not sustain prolonged transmission in Singapore. The Singaporean DENV3/III-C3 strains recovered between 2013 and 2014 were clustered within the Malaysian monophyletic group, suggesting that the later Singapore strains were probably reintroduced into Singapore from Malaysia, and which in turn was transmitted to the Saudi Arabia strain in 2014. Contemporaneous strains recovered from Thailand in 2010 and Taiwan in 2011 were also introduced from Malaysia, further supporting the widespread dispersal of the Malaysian DENV3/III-C. Collectively, the findings herein suggest and support the previous finding by Lee et al. [7] that the DENV3/III-C viruses were introduced into this region through a single event from the country (Sri Lanka?) where the virus has gone unsampled. Multiple independent dissemination events then contributed to the DENV3/III-C regional spread after 2008.

## Conclusions

Phylogenetic and spatiotemporal virus distribution analysis suggest that the recently circulating Malaysian DENV3/III was not the direct descendant of the old DENV3/III recovered in the 1980s. The isolates clustered with other isolates recovered contemporaneously from the neighboring countries and formed a monophyletic group in the phylogenetic tree that so far is restricted to isolates from Asia. Our findings suggest that the DENV3/III may have spread into Malaysia through multiple independent introduction events during the past 30 years. It is likely that Malaysia contributes to the spread of the recent DENV3/III-C3 in the Asian region in the early part of the 2010s. Only DENV3/III-C3, however, was successful in establishing a local transmission cycle in Malaysia and other South Eastern part of Asia. Factors that could contribute to its establishment, however, remained unclear. Moreover, it remained to be seen if the DENV3/III-C3 would be able to expand its geographical spread beyond Asia or remained geographically-restricted. Overall, our findings enhanced our understanding of DENV3/III diversity and its possible spread in different geographical context. Further study is needed to investigate the possible factor(s) that drive the cosmopolitan and non-cosmopolitan characteristics among these highly similar DENV3/III strains.

## Methods

### Sample preparation, genome sequencing, and assembly

All laboratory procedures involving the DENV isolates were performed in Biosafety Level 2 (BSL-2) laboratory following BSL-2 biosafety practices and procedures. The DENV3 isolates were obtained from the WHO Collaborating Centre for Arbovirus Reference & Research (Dengue/Severe Dengue) Virus Repository at University of Malaya (UM). Viral RNA was extracted from the supernatant of the DENV infected cell culture using QIAamp viral RNA mini kit (Qiagen, Germany) as previously described [41]. Full genome sequencing was done on either Applied Biosystems 3730xl DNA Analyzer (Life Technologies, USA) [41] or Ion Torrent sequencing platform (Life Technologies, USA) as previously described [42]. The sequencing data generated from Applied Biosystem 3730xl was analyzed and edited using Sequencher® v5.1 (Gene Code Corp, USA) [43]. Whereas, the raw sequencing reads generated from Ion Torrent sequencing platform were assembled using Genomics Short-read Nucleotide Alignment Program, GSNAP [44], integrated into Sequencher® v5.2.4 [45].

### Multiple sequence alignment, variant calling, and parsimony sites analysis

Multiple sequence alignment (MSA) of the Malaysian DENV3/III isolates along with DENV3/III sequences downloaded from GenBank were aligned using ClustalX 2.1 [46], resulting in datasets consisting 602 DENV3/III full genome sequences of virus strains recovered from 24 countries and 972 DENV3/III E gene sequences of virus strains recovered from 56 countries. Variant informative sites were extracted from the MSA of DENV3/III full genome using MEGA 6.0 [47]. Parsimony informative site was defined as variants that occurred with a frequency of at least two. The parsimony informative sites were arranged and sorted according to the order of DENV3/III on the MCC tree. Sequential amino acid substitution along the ORF-MCC tree was recorded and integrated into the ORF-MCC tree.

### Screening for intragenotypic recombination

The putative intragenotypic recombination event of DENV3/III within the ORF and E sequences datasets was screened using RDP4 [48]. Overall, the default setting with minor modification [49] where the highest acceptable *p*-value was adjusted to 0.01, was used for each algorithm during screening. Only the recombination event was concurrently identified by six or more algorithms was identified as recombination [49].

### Selection pressure analysis

The MSA of individual protein-coding gene from the entire virus genome: capsid (C), Pre-membrane (PrM), envelope (E), non-structural protein 1 (NS1), non-structural protein 2a (NS2a), non-structural protein 2b (NS2b), non-structural protein 3 (NS3), non-structural protein 4a (NS4a), non-structural protein 4b (NS4b), and non-structural protein 5 (NS5) were analyzed using HyPhy package [50] as implemented in the Datamonkey server [24]. Each MSA dataset was checked for duplication of genome sequences. The duplicated sequences were removed prior to the analysis. The specific site selection was analyzed using single likelihood ancestor counting (SLAC) [51], fixed effects likelihood (FEL) [51], internal fixed effects likelihood (IFEL) [52], Fast Unconstrained Bayesian AppRoximation (FUBAR) [53], and mixed effects model of evolution (MEME) [54] methods. Positive selection was defined as *p*-value ≤0.05 for SLAC, FEL, IFEL, and MEME. For FUBAR, the posterior probability of ≥0.9 was used as the cut-off value for positive selection as the analysis had relatively low false positive rate [53].

### Phylogenetic analysis

The sequences of the full coding region (10,170 bp) and E gene (2415 bp) were extracted from the MSA of DENV3/III strains. They were used as the input for the reconstruction of ORF and E phylogenetic tree, respectively. The phylogeny and the divergence time (tMRCA) of DENV3/III were estimated simultaneously using Bayesian Markov Chain Monte Carlo (MCMC) approach as implemented in BEAST 2.3 [55]. The Generalised time-reversible model with gamma distribution and invariant site (GTR + G + I) was selected using the Akaike Information Criterion (AIC) as implemented in jModel Test 2.1.4 [56]. The analysis was carried out under strict molecular clock model with MCMC chain length of 100 million, sampling every 10,000 generations. The resulting trace file was accessed using Tracer v1.6 [57]. The resulting trees were summarized into Maximum clade credibility (MCC) tree using TreeAnnotator V1.8.0 [58] and visualized using FigTree V1.4.2 [59]. The statistical significance of the tree nodes was determined using the posterior probability value.

### Phylogeographic analysis of DENV3 genotype III lineage C

In order to explore the phylogeography of DENV3/III-C, the E sequences of all DENV3/III-C (*n* = 102) were extracted from the initial E gene dataset. The 102 DENV3/III-C E gene sequences were used as the input for the reconstruction of a DENV3/III-C MCC tree using Beast 2.4.3 [55]. Phylogeographic analysis was performed with TrN93 model with gamma distribution (TrN93 + G), and a relaxed uncorrelated lognormal clock [60]. We used country of isolation of the DENV3/III-C strains over discrete diffusion model to reconstruct the possible ancestral location states of each internal branch. The analysis was performed under strict molecular clock model with MCMC chain length of 20 million, sampling every 2000 states.

## Additional files

Additional file 1: **Table S1.** Dengue virus type 3 strains used in analysis in this study. (DOCX 44 kb)

Additional file 2: Phylogeography of DENV3/III-C. (KML 1198 kb)

## Abbreviations

AIC: Akaike information criterion
BSL-2: Biosafety level 2
C: Capsid
DENV: Dengue virus
DENV1: Dengue virus type 1
DENV2: Dengue virus type 2
DENV3: Dengue virus type 3
DENV3/III: Dengue virus type 3 genotype III
DENV3/III-A: DENV3/III lineage A
DENV3/III-B: DENV3/III lineage B
DENV3/III-C: DENV3/III lineage C
DENV3/III-D: DENV3/III lineage D
DENV4: Dengue virus type 4
E: Envelope
FEL: Fixed effects likelihood
FUBAR: Fast unconstrained Bayesian AppRoximation
IFEL: Internal fixed effects likelihood
MCC: Maximum clade credibility
MEME: Mixed effects model of evolution
MSA: Multiple sequence alignment
NS1: Non-structural protein 1
NS2a: Non-structural protein 2
NS2b: Non-structural protein 2b
NS3: Non-structural protein 3
NS4a: Non-structural protein 4a
NS4b: Non-structural protein 4b
NS5: Non-structural protein 5
PrM: Pre-membrane
SLAC: Single likelihood ancestor counting
UM: University of Malaya
