# Supplementary material for: Emergence of the Asian lineage dengue virus type 3 genotype III in Malaysia
Source: BMC Evol Biol. 2018 Apr 24;18:58. doi: 10.1186/s12862-018-1175-4 (PMC5921268; doi:10.1186/s12862-018-1175-4)
Supplement: Supplementary file 1 — Table S1. Dengue virus type 3 strains used in analysis in this study. (DOCX 44 kb) [file 12862_2018_1175_MOESM1_ESM.docx]

Additional file

Table S1. List of Dengue virus type 3 strains used in analysis in this study

| Name | Origin | Year | Accession Number | Geographical region |
| --- | --- | --- | --- | --- |
| AI.BID-V2976/2001 | Anguilla | 2001 | FJ898462.1 | Caribbean |
| AU.Townsville_2007/2007 | Australia | 2007 | JN575578.1 | Oceania |
| BR.AL95/2009 | Brazil: Ribeirao Preto (Sao Paulo) | 2009 | JF808120.1 | South America |
| BR.BID-V2387/2003 | Brazil: Northern | 2003 | FJ850079.1 | South America |
| BR.BID-V2391/2004 | Brazil: Northern | 2004 | FJ850083.1 | South America |
| BR.BID-V2397/2006 | Brazil: Northern | 2006 | FJ850089.1 | South America |
| BR.BID-V2400/2007 | Brazil: Northern | 2007 | FJ850092.1 | South America |
| BR.BID-V2403/2008 | Brazil: Northern | 2008 | FJ850094.1 | South America |
| BR.BID-V3417/2006 | Brazil: Sao Paulo | 2006 | GU131844.1 | South America |
| BR.BID-V3456/2006 | Brazil: Sao Paulo | 2006 | GU131856.1 | South America |
| BR.BID-V3601/2007 | Brazil: Sao Paulo | 2007 | GU131874.1 | South America |
| BR.BID-V3615/2007 | Brazil: Sao Paulo | 2007 | GU131878.1 | South America |
| BR.BR73354mosq/2001 | Brazil | 2001 | FJ177308.1 | South America |
| BR.MR9/2003 | Brazil: Marituba (Para) | 2003 | JF808124.1 | South America |
| BR.SL3/2002 | Brazil: Sao Luis (Maranhao) | 2002 | JF808125.1 | South America |
| CN.13GDZDVS30B/2013 | China: Zhongshan | 2013 | KF954946.1 | East China |
| CN.13GDZDVS30C/2013 | China: Zhongshan | 2013 | KF954947.1 | East China |
| CN.13GDZSDV30A/2013 | China: Zhongshan | 2013 | KF954945.1 | East China |
| CN.GZ1D3/2009 | China: Guangzhou | 2009 | GU363549.1 | East China |
| CN.GZ2D3/2009 | China: Guangzhou | 2009 | JN662391.1 | East China |
| CN.ZJYW2009/2009 | China: Chejiang Yi Wu | 2009 | JF504679.1 | East China |
| CO.BID-V3394/2003 | Colombia: Santander | 2003 | GU131951.1 | South America |
| CO.BID-V3403/2005 | Colombia: Norte de Santander | 2005 | GQ868577.1 | South America |
| CO.BID-V3405/2007 | Colombia: Santander | 2007 | GQ868578.1 | South America |
| EC.BID-V2975/2000 | Ecuador | 2000 | FJ898457.1 | South America |
| GD.BID-V3930/2002 | Grenada | 2002 | KF955505.1 | Caribbean |
| GY.BID-V2980/2002 | Guyana | 2002 | FJ898464.1 | South America |
| IN.1416/1984 | India | 1984 | L11424 | South Asia |
| IN.DEL-72/2008 | India: Delhi | 2008 | GQ466079.1 | South Asia |
| IN.EHI0235/2007 | Indian subcontinent | 2007 | JN022604.1 | South Asia |
| IN.GWL-25/2003 | India: Gwalior | 2003 | AY770511.2 | South Asia |
| IN.ND143/2007 | India: Hyderabad | 2007 | FJ644564.1 | South Asia |
| IN.NIV_09401/2009 | India | 2009 | JQ686083.1 | South Asia |
| IN.NIV_664481/1966 | India | 1966 | JQ686068.1 | South Asia |
| IN.NIV_664482/1966 | India | 1966 | JQ686069.1 | South Asia |
| LC.BID-V2979/2001 | Saint Lucia | 2001 | FJ898463.1 | Caribbean |
| LC.BID-V3929/2001 | Saint Lucia | 2001 | GQ868616.1 | Caribbean |
| LK.3010/1993 | Sri Lanka | 1993 | KX518574.1 | South Asia |
| LK.3050/2004 | Sri Lanka | 2004 | KX518576.1 | South Asia |
| LK.3053/2004 | Sri Lanka | 2004 | KX518577.1 | South Asia |
| LK.3054/2004 | Sri Lanka | 2004 | KX518578.1 | South Asia |
| LK.3055/2003 | Sri Lanka | 2003 | KX518579.1 | South Asia |
| LK.3404/2006 | Sri Lanka (Germany) | 2006 | KU509283.1 | South Asia |
| LK.BID-V2404/1989 | Sri Lanka | 1989 | KF955474.1 | South Asia |
| LK.BID-V2405/1983 | Sri Lanka: Western Province | 1983 | GQ199887.1 | South Asia |
| LK.BID-V2407/1983 | Sri Lanka: Western Province | 1983 | GQ199888.1 | South Asia |
| LK.BID-V2409/1997 | Sri Lanka: Western Province | 1997 | GQ252674.1 | South Asia |
| LK.BID-V2410/1983 | Sri Lanka: Western Province | 1983 | GQ199889.1 | South Asia |
| LK.BID-V2411/1989 | Sri Lanka | 1989 | FJ882571.1 | South Asia |
| LK.BID-V2412/1989 | Sri Lanka | 1989 | FJ882572.1 | South Asia |
| LK.BID-V2413/1993 | Sri Lanka | 1993 | FJ882573.1 | South Asia |
| LK.BID-V2414/1985 | Sri Lanka | 1985 | FJ882574.1 | South Asia |
| LK.H_IMTSSA-SRI_1266/2000 | Sri Lanka | 2000 | AY099336.1 | South Asia |
| LK.SriLan9912aTw/1999 | Sri Lanka | 1999 | DQ518679.1 | South Asia |
| LK.UNC3001/1989 | Sri Lanka | 1989 | JQ411814.1 | South Asia |
| MQ.H_IMTSSA-MART_1243/1999 | Martinique | 1999 | AY099337.1 | Caribbean |
| MX.BID-V2985/2003 | Mexico: Morelos | 2003 | FJ898440.1 | North America |
| MX.BID-V2987/2006 | Mexico: Distrito Federal | 2006 | FJ898441.1 | North America |
| MX.BID-V2989/2007 | Mexico: Puebla | 2007 | FJ898442.1 | North America |
| MY.0811aTw/2008 | Malaysia | 2008 | JF968068.1 | Southeast Asia |
| MY.1009aTw/2010 | Malaysia | 2010 | JF968107.1 | Southeast Asia |
| MY.1012aTw/2010 | Malaysia | 2010 | JF968111.1 | Southeast Asia |
| MY.122290889/2010 | Malaysia | 2010 | in this study | Southeast Asia |
| MY.1308aTw/2013 | Malaysia (Taiwan) | 2013 | KP176709.1 | Southeast Asia |
| MY.1708603/2007 | Malaysia | 2007 | in this study | Southeast Asia |
| MY.1940004/2008 | Malaysia | 2008 | in this study | Southeast Asia |
| MY.2022453/2008 | Malaysia | 2008 | in this study | Southeast Asia |
| MY.2024403/2008 | Malaysia | 2008 | in this study | Southeast Asia |
| MY.2030755/2008 | Malaysia | 2008 | in this study | Southeast Asia |
| MY.2044396/2008 | Malaysia | 2008 | in this study | Southeast Asia |
| MY.2343777/2011 | Malaysia | 2011 | in this study | Southeast Asia |
| MY.2347048/2011 | Malaysia | 2011 | in this study | Southeast Asia |
| MY.2353394/2011 | Malaysia | 2011 | in this study | Southeast Asia |
| MY.2424900/2011 | Malaysia | 2011 | in this study | Southeast Asia |
| MY.2771163/2010 | Malaysia | 2010 | in this study | Southeast Asia |
| MY.2772324/2010 | Malaysia | 2010 | in this study | Southeast Asia |
| MY.2829147/2010 | Malaysia | 2010 | in this study | Southeast Asia |
| MY.2849608/2010 | Malaysia | 2010 | in this study | Southeast Asia |
| MY.2933228/2010 | Malaysia | 2010 | in this study | Southeast Asia |
| MY.3034309/2010 | Malaysia | 2010 | in this study | Southeast Asia |
| MY.3104570/2010 | Malaysia | 2010 | in this study | Southeast Asia |
| MY.3116826/2010 | Malaysia | 2010 | in this study | Southeast Asia |
| MY.3128939/2010 | Malaysia | 2010 | in this study | Southeast Asia |
| MY.59538/1987 | Malaysia | 1987 | in this study | Southeast Asia |
| MZ.BID-V2418/1985 | Mozambique | 1985 | FJ882575.1 | Eastern Africa |
| NI.BID-V2419/1998 | Nicaragua | 1998 | GQ199886.1 | Central America |
| NI.BID-V2420/1994 | Nicaragua | 1994 | FJ882576.1 | Central America |
| NI.BID-V2647/2008 | Nicaragua: Managua | 2008 | FJ873812.1 | Central America |
| NI.BID-V2653/2008 | Nicaragua: Managua | 2008 | FJ850048.1 | Central America |
| NI.BID-V2935/2008 | Nicaragua: Managua | 2008 | FJ898475.1 | Central America |
| NI.BID-V3169/2008 | Nicaragua: Managua | 2008 | HQ541790.1 | Central America |
| NI.BID-V4860/2009 | Nicaragua: Managua | 2009 | HQ705610.1 | Central America |
| NI.BID-V5095/2009 | Nicaragua: Managua | 2009 | JF920398.1 | Central America |
| NI.BID-V5680/2010 | Nicaragua: Managua | 2010 | JF937639.1 | Central America |
| NI.BID-V7646/2012 | Nicaragua | 2012 | KF973478.1 | Central America |
| NI.BID-V7658/2012 | Nicaragua | 2012 | KF973480.1 | Central America |
| NI.BID-V7665/2011 | Nicaragua | 2011 | KF973483.1 | Central America |
| NI.BID-V7694/2012 | Nicaragua | 2012 | KF973486.1 | Central America |
| NI.BID-V7699/2011 | Nicaragua | 2011 | KF973487.1 | Central America |
| PE.BID-V2981/2002 | Peru | 2002 | FJ898458.1 | South America |
| PE.BID-V6158/2002 | Peru | 2002 | KJ189256.1 | South America |
| PE.BID-V6170/2002 | Peru | 2002 | KJ189260.1 | South America |
| PE.BID-V6263/2008 | Peru | 2008 | KJ189261.1 | South America |
| PE.BID-V7041/2004 | Peru | 2004 | KJ189262.1 | South America |
| PE.BID-V7048/2004 | Peru | 2004 | KJ189266.1 | South America |
| PE.BID-V7056/2007 | Peru | 2007 | KJ189272.1 | South America |
| PE.BID-V7066/2007 | Peru | 2007 | KJ189282.1 | South America |
| PE.BID-V7071/2008 | Peru | 2008 | KJ189287.1 | South America |
| PE.BID-V7081/2009 | Peru | 2009 | KJ189292.1 | South America |
| PE.BID-V7083/2006 | Peru | 2006 | KJ189294.1 | South America |
| PE.BID-V7085/2006 | Peru | 2006 | KJ189296.1 | South America |
| PE.BID-V7086/2006 | Peru | 2006 | KJ189297.1 | South America |
| PK.43298/2006 | Pakistan: Karachi | 2006 | KF041259.1 | South Asia |
| PK.45251/2009 | Pakistan: Karachi | 2009 | KF041258.1 | South Asia |
| PK.52440/2006 | Pakistan: Karachi | 2006 | KF041257.1 | South Asia |
| PK.55505/2007 | Pakistan: Hyderabad | 2007 | KF041255.1 | South Asia |
| PK.55709/2006 | Pakistan: Karachi | 2006 | KF041256.1 | South Asia |
| PK.56/2008 | Pakistan: Karachi | 2008 | KF041254.1 | South Asia |
| PR.BID-V1043/2006 | USA: Puerto Rico | 2006 | EU482555.1 | Caribbean |
| PR.BID-V1044/2006 | USA: Puerto Rico | 2006 | EU529692.1 | Caribbean |
| PR.BID-V1076/1999 | USA: Puerto Rico | 1999 | EU529696.1 | Caribbean |
| PR.BID-V1078/2003 | USA: Puerto Rico | 2003 | EU482564.1 | Caribbean |
| PR.BID-V1080/2006 | USA: Puerto Rico | 2006 | EU529699.1 | Caribbean |
| PR.BID-V1090/1998 | USA: Puerto Rico | 1998 | EU529703.1 | Caribbean |
| PR.BID-V1091/2004 | USA: Puerto Rico | 2004 | EU529704.1 | Caribbean |
| PR.BID-V1416/2007 | USA: Puerto Rico | 2007 | EU596493.1 | Caribbean |
| PR.BID-V1417/2007 | USA: Puerto Rico | 2007 | EU596494.1 | Caribbean |
| PR.BID-V1449/1998 | USA: Puerto Rico | 1998 | EU726772.1 | Caribbean |
| PR.BID-V1466/1999 | USA: Puerto Rico | 1999 | EU687226.1 | Caribbean |
| PR.BID-V1606/2004 | USA: Puerto Rico | 2004 | FJ024465.1 | Caribbean |
| PR.BID-V1611/2004 | USA: Puerto Rico | 2004 | FJ850056.1 | Caribbean |
| PR.BID-V1612/2004 | USA: Puerto Rico | 2004 | FJ024470.1 | Caribbean |
| PR.BID-V1613/2004 | USA: Puerto Rico | 2004 | FJ024471.1 | Caribbean |
| PR.BID-V1618/2005 | USA: Puerto Rico | 2005 | FJ182008.1 | Caribbean |
| PR.BID-V1619/2005 | USA: Puerto Rico | 2005 | FJ182009.1 | Caribbean |
| PR.BID-V1623/2005 | USA: Puerto Rico | 2005 | FJ182038.1 | Caribbean |
| PR.BID-V1626/2005 | USA: Puerto Rico | 2005 | FJ182041.1 | Caribbean |
| PR.BID-V1728/2006 | Puerto Rico | 2006 | KF955456.1 | Caribbean |
| PR.BID-V1731/2003 | USA: Puerto Rico | 2003 | FJ205870.1 | Caribbean |
| PR.BID-V2098/1999 | USA: Puerto Rico | 1999 | FJ547069.1 | Caribbean |
| PR.BID-V2099/1998 | USA: Puerto Rico | 1998 | FJ547070.1 | Caribbean |
| PR.BID-V2111/2000 | USA: Puerto Rico | 2000 | FJ547076.1 | Caribbean |
| PR.BID-V2117/2001 | USA: Puerto Rico | 2001 | FJ547081.1 | Caribbean |
| PR.BID-V2118/2001 | USA: Puerto Rico | 2001 | FJ547082.1 | Caribbean |
| PR.BID-V2119/2002 | USA: Puerto Rico | 2002 | FJ547083.1 | Caribbean |
| PR.BID-V2126/2006 | USA: Puerto Rico | 2006 | FJ547085.1 | Caribbean |
| PR.BID-V859/1998 | USA: Puerto Rico | 1998 | EU482596.1 | Caribbean |
| PY.AS12/2002 | Paraguay: Asuncion (Central) | 2002 | JF808123.1 | South America |
| PY.SUS/2003 | Paraguay: Asuncion (Central | 2003 | JF808122.1 | South America |
| SA.Jeddah/2014 | Saudi Arabia | 2014 | KJ830751.1 | Western Asia |
| SG.01975Y13/2013 | Singapore | 2013 | KX224291.1 | Southeast Asia |
| SG.0379Y09/2009 | Singapore | 2009 | JN030177.1 | Southeast Asia |
| SG.0420Y08/2008 | Singapore | 2008 | JN030174.1 | Southeast Asia |
| SG.04800Y14/2014 | Singapore | 2014 | KX224283.1 | Southeast Asia |
| SG.0573Y08/2008 | Singapore | 2008 | JN030171.1 | Southeast Asia |
| SG.05K2400DK1/2005 | Singapore | 2005 | EU081193.1 | Southeast Asia |
| SG.05K2406DK1/2005 | Singapore | 2005 | EU081194.1 | Southeast Asia |
| SG.05K2418DK1/2005 | Singapore | 2005 | EU081195.1 | Southeast Asia |
| SG.05K2899DK1/2005 | Singapore | 2005 | EU081196.1 | Southeast Asia |
| SG.05K2918DK1/2005 | Singapore | 2005 | EU081197.1 | Southeast Asia |
| SG.05K2933DK1/2005 | Singapore | 2005 | EU081198.1 | Southeast Asia |
| SG.05K3305DK1/2005 | Singapore | 2005 | EU081199.1 | Southeast Asia |
| SG.05K3312DK1/2005 | Singapore | 2005 | EU081200.1 | Southeast Asia |
| SG.05K3314DK1/2005 | Singapore | 2005 | EU081201.1 | Southeast Asia |
| SG.05K3316DK1/2005 | Singapore | 2005 | EU081202.1 | Southeast Asia |
| SG.05K3324DK1/2005 | Singapore | 2005 | EU081203.1 | Southeast Asia |
| SG.05K3325DK1/2005 | Singapore | 2005 | EU081204.1 | Southeast Asia |
| SG.05K3329DK1/2005 | Singapore | 2005 | EU081205.1 | Southeast Asia |
| SG.05K3887DK1/2005 | Singapore | 2005 | EU081206.1 | Southeast Asia |
| SG.05K3897DK1/2005 | Singapore | 2005 | EU081207.1 | Southeast Asia |
| SG.05K3900DK1/2005 | Singapore | 2005 | EU081208.1 | Southeast Asia |
| SG.05K3912DK1/2005 | Singapore | 2005 | EU081209.1 | Southeast Asia |
| SG.05K3913DK1/2005 | Singapore | 2005 | EU081210.1 | Southeast Asia |
| SG.05K3923DK1/2005 | Singapore | 2005 | EU081211.1 | Southeast Asia |
| SG.05K3927DK1/2005 | Singapore | 2005 | EU081212.1 | Southeast Asia |
| SG.05K3928DK1/2005 | Singapore | 2005 | EU081213.1 | Southeast Asia |
| SG.05K4141DK1/2005 | Singapore | 2005 | EU081214.1 | Southeast Asia |
| SG.05K4144DK1/2005 | Singapore | 2005 | EU081215.1 | Southeast Asia |
| SG.05K4157DK1/2005 | Singapore | 2005 | EU081216.1 | Southeast Asia |
| SG.05K4159DK1/2005 | Singapore | 2005 | EU081217.1 | Southeast Asia |
| SG.05K4168DK1/2005 | Singapore | 2005 | EU081218.1 | Southeast Asia |
| SG.05K4176DK1/2005 | Singapore | 2005 | EU081219.1 | Southeast Asia |
| SG.05K4182DK1/2005 | Singapore | 2005 | EU081220.1 | Southeast Asia |
| SG.05K4454DK1/2005 | Singapore | 2005 | EU081222.1 | Southeast Asia |
| SG.05K4647DK1/2005 | Singapore | 2005 | EU081224.1 | Southeast Asia |
| SG.05K4648DK1/2005 | Singapore | 2005 | EU081225.1 | Southeast Asia |
| SG.05K791DK1/2005 | Singapore | 2005 | EU081182.1 | Southeast Asia |
| SG.05K797DK1/2005 | Singapore | 2005 | EU081183.1 | Southeast Asia |
| SG.05K802DK1/2005 | Singapore | 2005 | EU081184.1 | Southeast Asia |
| SG.05K805DK1/2005 | Singapore | 2005 | EU081185.1 | Southeast Asia |
| SG.05K827DK1/2005 | Singapore | 2005 | EU081186.1 | Southeast Asia |
| SG.05K843DK1/2005 | Singapore | 2005 | EU081187.1 | Southeast Asia |
| SG.05K845DK1/2005 | Singapore | 2005 | EU081188.1 | Southeast Asia |
| SG.05K852DK1/2005 | Singapore | 2005 | EU081189.1 | Southeast Asia |
| SG.05K863DK1/2005 | Singapore | 2005 | EU081190.1 | Southeast Asia |
| SG.05K868DK1/2005 | Singapore | 2005 | EU081191.1 | Southeast Asia |
| SG.05K871DK1/2005 | Singapore | 2005 | EU081192.1 | Southeast Asia |
| SG.0624Y08/2008 | Singapore | 2008 | JN030175.1 | Southeast Asia |
| SG.0673Y08/2008 | Singapore | 2008 | JN030165.1 | Southeast Asia |
| SG.06995Y09/2009 | Singapore | 2009 | JN030184.1 | Southeast Asia |
| SG.0842Y08/2008 | Singapore | 2008 | JN030168.1 | Southeast Asia |
| SG.1358Y08/2008 | Singapore | 2008 | JN030185.1 | Southeast Asia |
| SG.1392Y08/2008 | Singapore | 2008 | JN030187.1 | Southeast Asia |
| SG.14856Y13/2013 | Singapore | 2013 | KX224292.1 | Southeast Asia |
| SG.1621Y08/2008 | Singapore | 2008 | JN030166.1 | Southeast Asia |
| SG.16603Y13/2013 | Singapore | 2013 | KR779787.1 | Southeast Asia |
| SG.26592Y13/2013 | Singapore | 2013 | KP685235.1 | Southeast Asia |
| SG.38213Y13/2013 | Singapore | 2013 | KX224280.1 | Southeast Asia |
| SG.40588Y10/2010 | Singapore | 2010 | JN030189.1 | Southeast Asia |
| SG.47593Y09/2009 | Singapore | 2009 | JN030180.1 | Southeast Asia |
| SG.SS710/2004 | Singapore | 2004 | EU081181.1 | Southeast Asia |
| TH.1009aTw/2010 | Thailand | 2010 | JF968106.1 | Southeast Asia |
| TT.BID-V2982/2002 | Trinidad and Tobago | 2002 | FJ898459.1 | South America |
| TT.BID-V3928/2002 | Trinidad and Tobago: Trinidad | 2002 | GQ868617.1 | South America |
| TW.928PT1111a/2011 | Taiwan | 2011 | KP176714.1 | East Asia |
| TW.99TW628/1999 | Taiwan | 1999 | DQ675533.1 | East Asia |
| VE.BID-V1102/2007 | Venezuela: Aragua | 2007 | EU529683.1 | South America |
| VE.BID-V1593/2005 | Venezuela: District Federal Caracas | 2005 | EU854292.1 | South America |
| VE.BID-V2198/2001 | Venezuela: Aragua | 2001 | FJ639768.1 | South America |
| VE.BID-V2210/2002 | Venezuela: Aragua | 2002 | FJ639777.1 | South America |
| VE.BID-V2219/2003 | Venezuela: Aragua | 2003 | FJ639786.1 | South America |
| VE.BID-V2231/2004 | Venezuela: Aragua | 2004 | FJ639798.1 | South America |
| VE.BID-V2267/2008 | Venezuela: Aragua | 2008 | FJ639826.1 | South America |
| VE.BID-V2268/2008 | Venezuela: Aragua | 2008 | FJ639827.1 | South America |
| VE.BID-V2482/2007 | Venezuela: Aragua | 2007 | FJ850110.1 | South America |
| VE.BID-V2971/2007 | Venezuela: Aragua | 2007 | FJ898474.1 | South America |
| VE.BID-V903/2001 | Venezuela: Caracas | 2001 | EU529688.1 | South America |
| VE.BID-V911/2001 | Venezuela: Caracas | 2001 | EU529691.1 | South America |
| WS.1696/1986 | Samoa | 1986 | L11435 | Oceania |
